# Effect of Ethanol Vapor Treatment on the Growth of *Alternaria alternata* and *Botrytis cinerea* and Defense-Related Enzymes of Fungi-Inoculated Blueberry During Storage

**DOI:** 10.3389/fmicb.2021.618252

**Published:** 2021-01-26

**Authors:** Yaru Ji, Wenzhong Hu, Jia Liao, Zhilong Xiu, Aili Jiang, Xiaozhe Yang, Yuge Guan, Ke Feng, Gaowa Saren

**Affiliations:** ^1^School of Bioengineering, Dalian University of Technology, Dalian, China; ^2^Key Laboratory of Biotechnology and Bioresources Utilization, Ministry of Education, Dalian, China; ^3^College of Life Science, Dalian Minzu University, Dalian, China

**Keywords:** blueberry, ethanol vapor, Alternaria alternata, Botrytis cinerea, defense-related enzymes

## Abstract

The aim of the present study was to investigate the effects of ethanol vapor on the inhibition of *Alternaria alternata* and *Botrytis cinerea* in postharvest blueberry and the induction of defense-related enzymes (DREs) activities in fungi-inoculated blueberries stored at 0±0.5°C for 16days. Results indicated that ethanol vapor markedly inhibited the mycelial growth of *A. alternata* and *B. cinerea* in a dose-dependent manner, with inhibition rates of 9.1% (250μlL^−1^), 36.4% (500μlL^−1^), and 5.5% (1,000μlL^−1^) on *A. alternata* and 14.2% (250μlL^−1^), 44.7% (500μlL^−1^), and 76.6% (1,000μlL^−1^) on *B. cinerea*, respectively. Meanwhile, ethanol vapor also enhanced the activities of DREs in fungi-inoculated blueberries, including *β*-1,3-glucanase (GLU), chitinase (CHI), phenylalnine ammonialyase (PAL), peroxidase (POD), and polyphenol oxidase (PPO). In particular, 500μlL^−1^ ethanol vapor increased the activities of DREs by 84.7% (GLU), 88.0% (CHI), 37.9% (PAL), 85.5% (POD), and 247.0% (PPO) in *A. alternata*-inoculated blueberries and 103.8% (GLU), 271.1% (CHI), 41.1% (PAL), 148.3% (POD), and 74.4% (PPO) in *B. cinerea*-inoculated blueberries, respectively. But, the activity of PPO was decreased by 55.2 and 31.9% in 500μlL^−1^ ethanol-treated blueberries inoculated with *A. alternata* and *B. cinerea*, respectively, after 8days of storage. Moreover, the surface structure and ultrastructure of 500μlL^−1^ ethanol-treated blueberry fruit cells were more integrated than those of other treatments. The findings of the present study suggest that ethanol could be used as an activator of defense responses in blueberry against *Alternaria* and *Botrytis* rots, by activating DREs, having practical application value in the preservation of postharvest fruit and vegetables.

## Introduction

Blueberries (*Vaccinium* spp., Ericaceae) are native to North America, but are cultivated worldwide, and are an increasingly popular functional fruit, with production increasing by 60.7% between 2010 and 2019 (FAO, 2020). The nutritional value of the fruit can be attributed to its remarkably high levels of health-promoting components, including anthocyanins, vitamins, flavonols, and dietary fiber (Wang et al., 2012; Chen et al., 2017). However, fresh blueberries are highly perishable, owing to senescence caused by physiological metabolism, physical damage (e.g., mechanical damage and temperature effects), and decay caused by microbial pathogens (Ji et al., 2019), and microbial infection is the main factor that limits blueberry storage, transportation, and marketing. Furthermore, the aging and softening of blueberries during storage weaken the fruit’s resistance to pathogens that lurk on the fruit surface and in the environment. The fungal pathogens that cause blueberry rot differ by area, but the principal pathogenic fungi include *Alternaria alternata* (Alternaria rot), *Botrytis cinerea* (Botrytis rot), and *Colletotrichum acutatum* (Anthracnose rot; Wang et al., 2010).

*Botrytis cinerea* (*B. cinerea*), one of the main rotting fungi, generally colonizes and infects senescent flower remnants of blueberries directly by secreting cell wall degrading enzymes (pectin lyase, polygalacturonase, etc.) and virulence factors (organic acids, botrydial, etc.) before harvest (Huang et al., 2012; Zhang et al., 2014), but can also infect blueberry fruit through stem scar and mechanical wounds, especially when the fruits’ resistance mechanisms are reduced after harvest. Furthermore, even though the optimum temperature for *B. cinerea* spore germination is 15–25°C, the pathogen can also grow at very low temperatures (e.g., −2°C), which enables it to affect fruit during cold storage (Sommer, 1985). During initial infection, the skin of *B. cinerea*-infected fruit is gray-white, and as soft rot occurs, numerous gray mycelia, which are rich in conidia, emerge on the surface of the diseased portion of the fruit, accompanied by slight fruit wilting. After air drying, the fruit is shriveled and stiff.

Meanwhile, *Alternaria* spp. are major pathogenic fungi of many fruit and vegetables, such as grapefruit, strawberry, grape, tomato, pepper, and onion, with *A. alternata* being the most damaging to blueberry fruit (Barkai, 2001; Wang et al., 2021). The optimum temperature for *A. alternata* spore germination is about 28°C. However, the pathogen can also maintain growth at −3°C, which enables it to maintain growth during cold chain transportation and refrigeration (Sommer, 1985). During infection, the mycelia of *A. alternata* penetrate the epidermis of blueberry fruit and then remain dormant in the fruit, only reactivating when the concentration of antifungal substances in the fruit decreases. Indeed, studies have shown that *A. alternata* is latent on blueberry fruit before harvest, and *A. alternata*-infected blueberry fruit is usually harvested together with healthy fruit, thereby spreading the disease throughout the postharvest chain, including picking, transportation, packaging, and storing. During initial infection, the pericarp of *A. alternata*-infected fruit exhibits sunken, dark-brown spots, and then numerous white mycelia grow on the diseased portion of the fruit (Caruso and Ramsdell, 1995).

Ethanol has long been used as a disinfectant, owing to its antimicrobial property, and, as a plant secondary metabolite, has low toxicity to fruit and vegetables. As such, ethanol is a FDA-certified generally recognized safe substance (GRAS) in the United States and a food industrial additive with unrestricted residues prescribed by GB 2760-2014 in China. Many studies have investigated the use of ethanol dip or vapor as non-biological control agent for reducing fruit decay and for extending the shelf-life of fruit and vegetables, such as sweet cherry (Karabulut et al., 2004a), Chinese bayberries (Wang et al., 2011), and table grapes (Lichter et al., 2002; Candir et al., 2012). In fact, ethanol can directly kill or inhibit the growth of rot microorganisms and may also be absorbed by the treated fruit or vegetables, thereby enhancing their disease resistance. Studies have reported that 30–40% ethanol dips can kill *B. cinerea* conidia isolated from table grapes *in vitro* (Lichter et al., 2002; Karabulut et al., 2004b), and that ethanol vapor can effectively reduce the postharvest decay rate of blueberry fruit (Ji et al., 2019). However, little information is available regarding the effects of ethanol treatment at a method of fumigation on the germination of *B. cinerea* and *A. alternata* spores or on the disease resistance of blueberry fruit.

Accordingly, the objectives of the present study were to investigate (1) the antifungal activities of ethanol vapor against *A. alternata* and *B. cinerea in vitro* and (2) the effect of ethanol treatment on the activities of defense-related enzymes in blueberry fruit. This research will contribute to the practical application of ethanol for preserving the postharvest fruit and vegetables.

## Materials and Methods

### Blueberry and Fungal Materials

Southern highbush blueberries (*Vaccinium corymbosum* L. “O’Neal”) used in this trial, were hand-harvested from the blueberry plantation at the Dalian Blueberry Technology Development Co., Ltd. facility in Zhuanghe City (Dalian, Liaoning, China) during the summer (June) of 2017 and 2018 (two repetitions). The fruits were consistent in maturity (100% blue) and transported to the laboratory within 3h of harvest using a refrigerated vehicle (10°C, S.F. Express, Dalian, Liaoning, China). In the laboratory, the fruits were cooled for 24h at 0±0.5°C and 90% RH, and then undamaged fruits that were uniform in size and color were selected (0±0.5°C and 90% RH) for use.

*Alternaria alternata* and *B. cinerea* were obtained from School of Bioengineering, Dalian University of Technology, China, and were maintained at 4°C in the dark in Petri dishes containing Potato Dextrose Agar (PDA; Hopebio, Qingdao, China).

### Spores Suspension Pretreatment

Both *A. alternata* and *B. cinerea* were cultured on PDA slants at 28°C, and after 5days, 5ml septic NaCl solution (0.9%, v/v) was added to each slant and slowly scraped off the agar surface. The aseptic NaCl solutions containing pathogen spores were each transferred to a sterile triangle bottle and then dispersed with glass beads. After shaking, the spore solutions were filtered using absorbent cotton to remove mycelial fragments and were then washed 2–3 times with aseptic NaCl solution. Finally, 1×10^6^CFUml^−1^ spore suspensions were prepared as needed using a blood cell counting board.

### Ethanol Vapor Treatment of *A. alternata* and *B. cinerea* on PDA

Melted PDA (15–20ml) was poured into a disposable plastic culture dish (90mm×20mm), and after coagulation, a 6-mm-diameter hole was dug in the medium in the center of each culture dish and inoculated with 20μl spore suspension. Sterile filter paper was trimmed to fit inside Petri dish covers, and according to the remaining space volume, absolute ethanol was dropped onto each filter paper-lined Petri dish cover, which was then placed quickly on its corresponding dish. Ethanol volumes were calculated to achieve concentrations of 0 (Control), 250, 500, or 1,000μlL^−1^ (ratio of liquid ethanol to container volume). The inoculated and treated plates were then sealed with preservative film, inverted, and incubated at 28°C. After 7days, the vertical diameters (mm) of the colonies were measured using a caliper, and inhibition rate was calculated as follows:

$$\mathrm{Inhibition rate}=\left(C-T\right)/C\times 100\%.$$

where *C* is the mean (*n*=3) colony diameter of the control group and *T* is the mean (*n*=3) colony diameter of the treatment group. Each treatment included three replicates, and the experiment was repeated three times.

### Ethanol Vapor Treatment of Blueberries Inoculated With *A. alternata* and *B. cinerea*

#### Inoculation

Groups of 50 individual fruit (400 fruit total) were superficially sterilized by a 2-min immersion in 2% (v/v) sodium hypochlorite solution, washed for 2min in sterile distilled, and then dried on sterile filter paper. The fruits were then wounded (2mm diameter×3mm deep) at the equator of each fruit with a sterilized nail and inoculated with 10μl either *A. alternata* (50 fruit×4) or *B. cinerea* (50 fruit×4) spore suspension (1.0×10^6^CFUml^−1^).

#### Ethanol Vapor Treatment

All inoculated blueberries were left to air-dry and then divided into eight groups: *A*-Control, *A*-250, *A*-500, *A*-1000, *B*-Control, *B*-250, *B*-500, and *B*-1000 (*A*-Control, inoculated with *A. alternata* and treated with 0μlL^−1^ ethanol; *A*-250, inoculated with *A. alternata* and treated with 250μlL^−1^ ethanol; *A*-500, inoculated with *A. alternata* and treated with 500μlL^−1^ ethanol; *A*-1000, inoculated with *A. alternata* and treated with 1,000μlL^−1^ ethanol; *B*-Control, inoculated with *B. cinerea* and treated with 0μlL^−1^ ethanol; *B*-250, inoculated with *B. cinerea* and treated with 250μlL^−1^ ethanol; *B*-500, inoculated with *B. cinerea* and treated with 500μlL^−1^ ethanol; and *B*-1000, inoculated with *B. cinerea* and treated with 1,000μlL^−1^ ethanol). More specifically, six disposable Petri dishes without cover that contained qualitative filter paper (to allow gradual diffusion of the ethanol), were placed in the bottom of each photosynthetic modified atmosphere preservation container (dimensions: 46.3cm×27.3cm×26.6cm; Beijing Hengqingyuan Technology Co. Ltd., Beijing, China), and the blueberries (with the inoculation wound facing up) were put on the hollowed-out interlayer of each container. Then, the blueberries in each group were subjected to ethanol vapor treatments with corresponding amount of ethanol (99.7%; 0, 250, 500, or 1,000μlL^−1^) dripped on the qualitative filter papers for 18h (0±0.5°C, 90% RH). Three containers were prepared for each treatment. After ethanol treatment, the blueberries were ventilated (0±0.5°C, 90% RH) for 24h, transferred to commercial PET plastic boxes (10.5cm×10.5cm×3cm), and then stored at 0±0.5°C and 90% RH. The treated and cold-stored blueberries were randomly sampled at different storage time points (0, 4, 8, 12, and 16days), frozen in liquid nitrogen, and then stored at −80°C until analysis. All the analyses were performed in triplicate, and this is a split-plot experimental design, with a 4×5 (doses×days) factorial design.

#### Determination of DREs Activities

The activities of *β*-1,3-glucanase (GLU), chitinase (CHI), phenylalnine ammonialyase (PAL), peroxidase (POD), and polyphenol oxidase (PPO) were determined using assay kits (Suzhou Keming Biotechnology Co., Ltd., Suzhou, Jiangsu, China).

Extraction of crude enzyme solution: according to the instructions of the kits, 0.15–0.2g of fruit sample was homogenized with 1ml of corresponding extraction solution in ice bath. After centrifugation at 10000×*g* for 20min at 4°C, the supernatants were collected and placed on ice for enzyme activity determination.

Then, the reagents were added in accordance with the instructions, and the absorbance values at 550 (GLU), 585 (CHI), 290 (PAL), 470 (POD), and 525 (PPO) nm were measured, respectively. Both the GLU and CHI activities were expressed as mg h^−1^ g^−1^. The activities of PAL, POD, and PPO were expressed as U g^−1^, where U represented a 0.1 change in absorbance (290nm), 0.005 change in absorbance (470nm), and 0.005 change in absorbance (525nm), respectively, per minute per gram of tissue per ml of the reaction system.

### Production and Observation of Electron Microscope Sections

Each group (*A*-Control, *A*-500, *A*-1000, *B*-Control, *B*-500, and *B*-1000) was randomly sampled at day 8 of the storage for scanning electron microscope (SEM) and transmission electron microscope (TEM) observation. Fresh picked blueberries were taken and treated with the same wound (2mm diameter×3mm deep), as the control without inoculation with rotting fungi (Wound-Control).

For SEM, the selected blueberry samples were finely cut into transparent slices using a surgical blade, immediately immersed in 2.5% (v/v) glutaraldehyde solution, and fixed overnight at 4°C. The following day, they were washed three times (5–10min each) using sterile distilled water, dehydrated using an ethanol gradient (15min each in 50, 70, 90, and 95% ethanol, v/v, and twice for 10–15min in 100% ethanol), soaked in isoamyl acetate (15min), and then dried at room temperature (~25°C). The dried samples were then adhered to aluminum discs using double-sided carbon tape, sputter-coated using gold, and then scanned and photographed using a Hitachi S-4800 SEM (Hitachi High-Technologies Co., Tokyo, Japan).

For TEM, an appropriate section (about 1mm×1mm×3mm) of flesh with peel was cut from each selected blueberry, immediately immersed in 2.5% (v/v) glutaraldehyde solution, and fixed overnight at 4°C. The following day, the fixed samples were soaked in 1% osmium tetroxide solution for 2h, washed three times in phosphate buffer solution (0.1M), dehydrated using an ethanol gradient solution (15min each in 30, 50, 70, 90, and 95% ethanol, v/v) and acetone (15min; 100%, v/v), embedded in epoxy resin (60°C for 72h), and then cut into ~70-nm slices using a Leica EM UC7 Ultramicrotome (Leica, Germany). The ultrathin sections were finally double-stained using uranyl acetate (25–30min) and lead citrate (5–10min) and then observed and filmed using a Hitachi JEM-2100 transmission electron microscope.

### Statistical Analysis

All the experiments were performed in triplicate. The bar charts were generated using Origin Software 8.0, and all data were analyzed using SPSS 14.0. One-way ANOVA and least significant difference (LSD) tests were used to differentiate mean values, and differences at the *p* <0.05 level were considered significant.

## Results

### Effect of Ethanol Vapor on Fungal Growth

**Figure 1:**
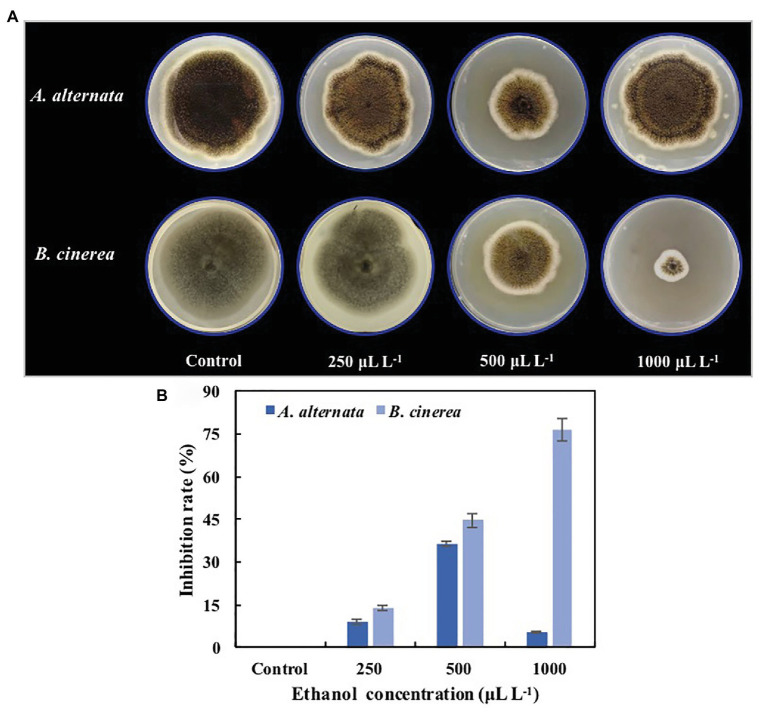
Inhibition effects of ethanol vapor on the growth of *Alternaria alternata* and *Botrytis cinerea*. **(A)** Visual effects of ethanol vapor treatments. **(B)** Inhibition rates of ethanol vapor treatments. Error bars represent the standard deviation of the means (*n*=3).

The colony diameters of both *A. alternata* and *B. cinerea* on PDA medium were significantly reduced by ethanol vapor treatment (Figure 1A), and the mycelial growth of *A. alternata* and *B. cinerea* was inhibited by 9.1 and 14.2% (250μlL^−1^), 36.4 and 44.7% (500μlL^−1^), and 5.5 and 76.6% (1,000μlL^−1^; Figure 1B). Although the inhibition rates of ethanol against the pathogens generally increased with increasing ethanol concentration, the ethanol treatments had a significantly greater inhibition effect on *B. cinerea* than on *A. alternata* (*p*<0.05), and the inhibition of *A. alternata* reached a maximum at 500μlL^−1^.

### Effect of Ethanol Vapor on DREs Activities in Blueberry

**Figure 2:**
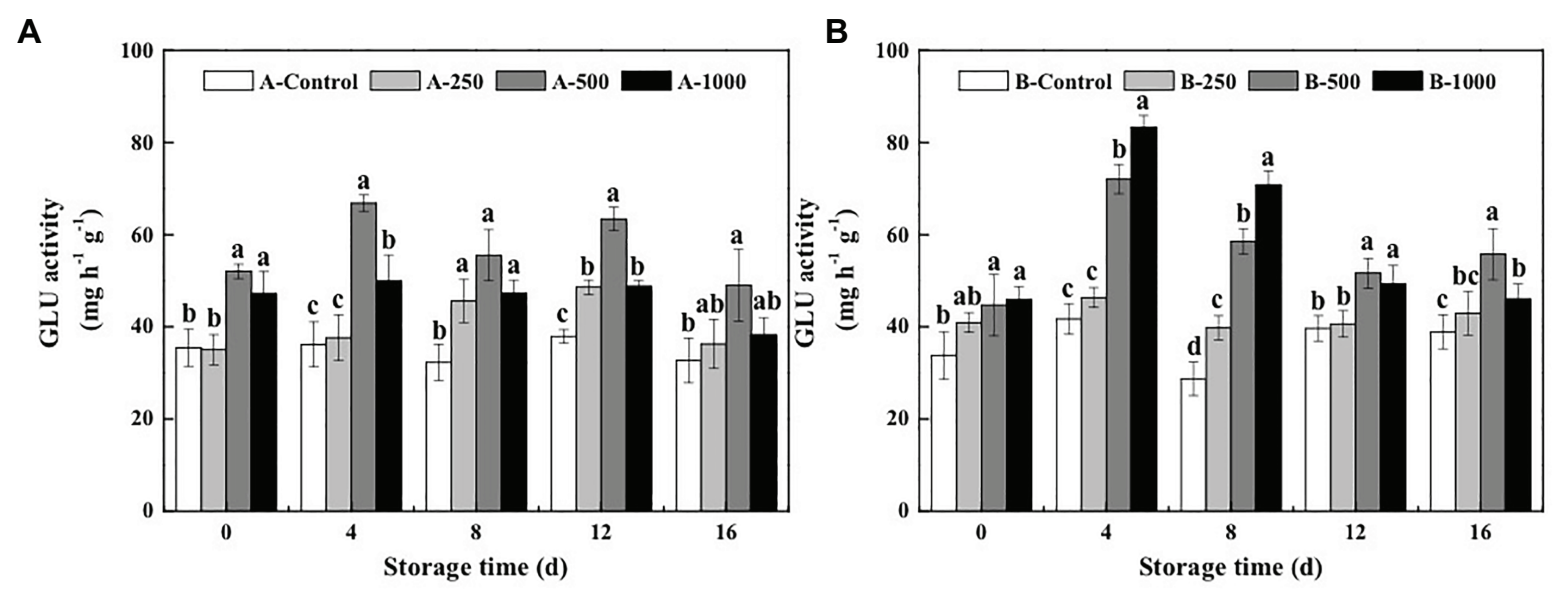
Effect of ethanol vapor on the *β*-1,3-glucanase (GLU) activity of blueberries inoculated with **A** (*A. alternata*, A) and **B** (*B. cinerea*, B). Two-hundred and fifty, 500, and 1,000 are the amount of ethanol used (μlL^−1^). Error bars represent the standard deviation of the means (*n*=3). Different letters on the same column represent significant differences (*p*<0.05).

**Figure 3:**
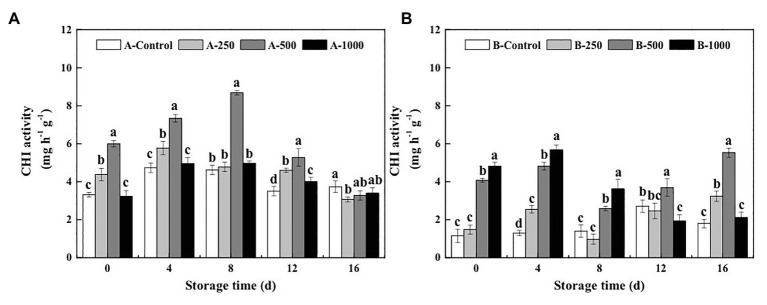
Effect of ethanol vapor on the chitinase (CHI) activity of blueberries inoculated with **A** (*A. alternata*, A) and **B** (*B. cinerea*, B). Two-hundred and fifty, 500, and 1,000 are the amount of ethanol used (μlL^−1^). Error bars represent the standard deviation of the means (*n*=3). Different letters on the same column represent significant differences (*p*<0.05).

The results of the present study demonstrate that both the GLU and CHI activities of *A. alternata*- or *B. cinerea*-inoculated blueberry fruit can be increased by treatment with appropriate concentrations of ethanol vapor (Figures 2, 3).

*β*-1,3-glucanase activity of the control inoculated blueberries was relatively constant during the 16-day storage period and was consistently lower than those of the ethanol vapor-treated inoculated blueberries (Figure 2). Furthermore, GLU activity of the *A. alternata*-inoculated blueberries that was treated with 500μlL^−1^ ethanol was significantly greater than those of the other treatment groups and reached a maximum of 66.83mgh^−1^ g^−1^ at day 4 of storage, which was 1.85, 1.78, and 1.34 times that of the *A. alternata*-inoculated blueberries treated with 0, 250, or 1,000μlL^−1^ ethanol, respectively (Figure 2A). However, a different pattern was observed for the blueberries inoculated with *B. cinerea* (Figure 2B). During the first 12days of storage, GLU activity of the 500 and 1,000μlL^−1^ ethanol-treated *B. cinerea*-inoculated blueberries was significantly greater than those of the control, and GLU activity of the 1,000μlL^−1^ ethanol-treated blueberries was better than that of the 500μlL^−1^ ethanol-treated blueberries. Similar to measurements of GLU activity in the *A. alternata*-inoculated blueberries, GLU activity of the *B. cinerea*-inoculated blueberries treated with 500 and 1,000μlL^−1^ ethanol reached maximum values (72.05 and 83.31mgh^−1^ g^−1^, respectively) at day 4 of storage, which were 1.73 and 2.00 times greater than that of the control, respectively.

Meanwhile, 250, 500, and 1,000μlL^−1^ ethanol treatment increased CHI activity of *A. alternata*-inoculated blueberries by 3–32, 55–88, and −3–7%, respectively, and increased those of *B. cinerea*-inoculated blueberries by −30–97, 85–271, and 159–337%, respectively (Figure 3). The maximum CHI activity (8.69mgh^−1^ g^−1^) of the *A. alternata*-inoculated blueberries was observed at day 8 after treatment with 500μlL^−1^ ethanol, which was later than that of the other groups (day 4), and the maximum activity of the 500μlL^−1^ ethanol-treated blueberries was 1.83, 1.50, and 1.75 times greater than that of the 0, 250, and 1,000μlL^−1^ ethanol-treated blueberries, respectively (Figure 3A). However, the maximum CHI activity (2.72mgh^−1^ g^−1^) of the *B. cinerea*-inoculated blueberries without ethanol treatment (B-Control) was observed on the 12th day of storage (Figure 3B). At the same time, the activity of 500μlL^−1^ ethanol-treated blueberries was 3.70mgh^−1^ g^−1^, which was 1.36 times higher than that in the B-Control.

**Figure 4:**
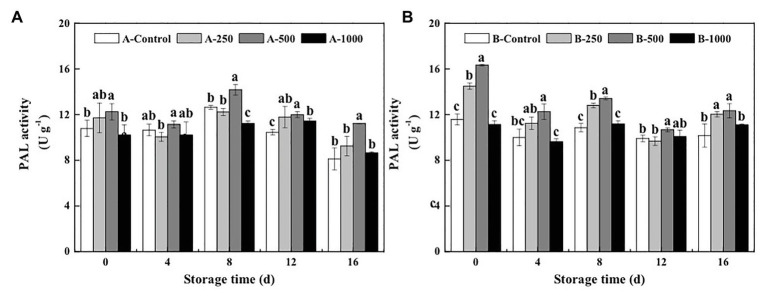
Effect of ethanol vapor on the phenylalnine ammonialyase (PAL) activity of blueberries inoculated with **A** (*A. alternata*, A) and **B** (*B. cinerea*, B). Two-hundred and fifty, 500, and 1,000 are the amount of ethanol used (μlL^−1^). Error bars represent the standard deviation of the means (*n*=3). Different letters on the same column represent significant differences (*p*<0.05).

Appropriate concentrations of ethanol vapor treatment had an immediate effect on PAL activity of blueberries inoculated with *A. alternata* and *B. cinerea* (Figure 4). PAL activity was activated quickly, and even at day 0 after treatment, the 500μlL^−1^ ethanol treatment increased PAL activity of the *A. alternata*- and *B. cinerea*-inoculated blueberries to 1.14 and 1.41 times higher than those of their control, respectively. Moreover, the 500μlL^−1^ ethanol treatment consistently induced the greatest activity level over the 16-day storage period, whereas the 1,000μlL^−1^ ethanol treatment had little effect. After 8days of storage, PAL activity of *A. alternata*- and *B. cinerea*-inoculated blueberries treated with 500μlL^−1^ ethanol was 1.12 and 1.24 times higher than those of the control, respectively.

**Figure 5:**
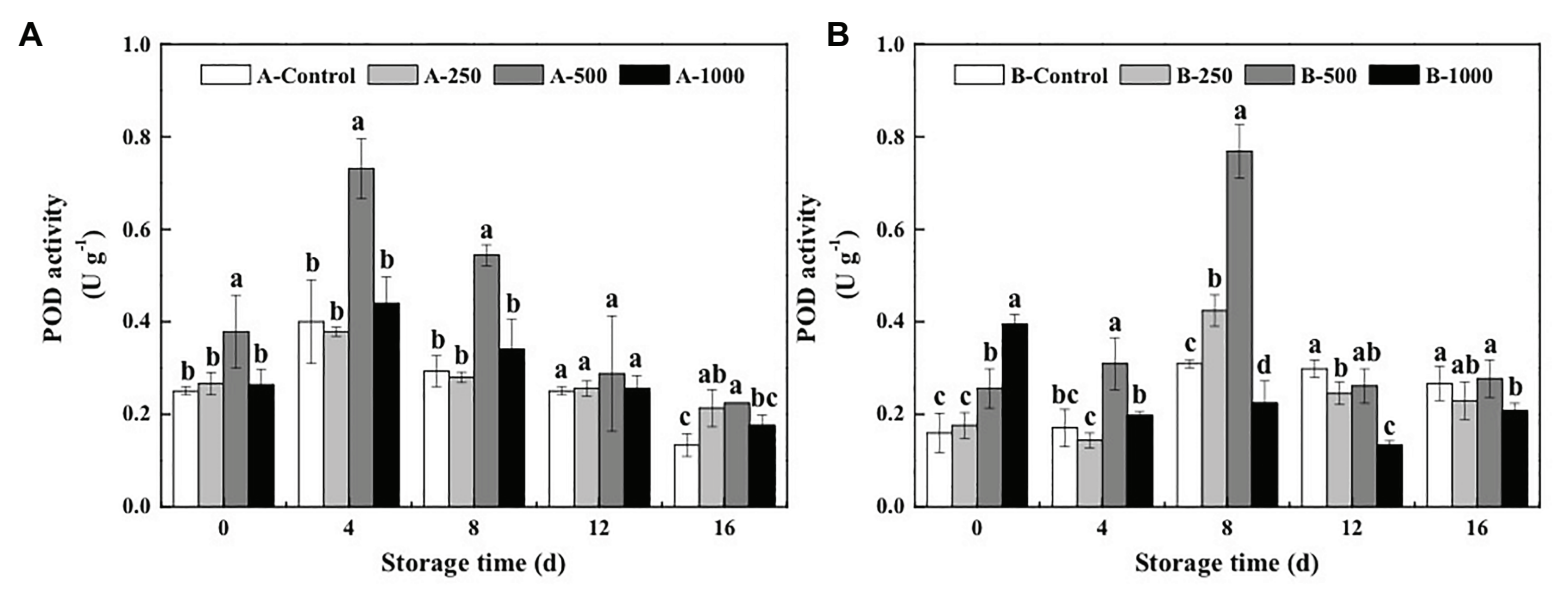
Effect of ethanol vapor on the peroxidase (POD) activity of blueberries inoculated with **A** (*A. alternata*, A) and **B** (*B. cinerea*, B). Two-hundred and fifty, 500, and 1,000 are the amount of ethanol used (μlL^−1^). Error bars represent the standard deviation of the means (*n*=3). Different letters on the same column represent significant differences (*p*<0.05).

As shown in Figure 5A, POD activity of all the *A. alternata*-inoculated blueberries exhibited the same trend, throughout the 16-day storage period, first increasing and then decreasing, and only the 500μlL^−1^ ethanol treatment significantly improved the POD activity of the *A. alternata*-inoculated blueberries, whereas treatments with lower (250μlL^−1^) and higher (1,000μlL^−1^) concentrations had no significant effect compared with the control (*p*>0.05). For all treatment groups, POD activities of blueberries reached a maximum on the 4th day of storage, at which point the POD activity of the *A. alternata*-inoculated blueberries treated with 500μlL^−1^ was 1.83, 1.93, and 1.66 times the activities of the other *A. alternata*-inoculated blueberries treated with 0, 250, and 1,000μlL^−1^, respectively. The POD activity of *B. cinerea*-inoculated blueberries was also increased significantly by the 500μlL^−1^ ethanol treatment but peaked on the 8th day of storage (Figure 5B), at which point the POD activity of the *B. cinerea*-inoculated blueberries treated with 500μlL^−1^ ethanol was 2.48, 1.81, and 3.43 times the activity of *B. cinerea*-inoculated blueberries treated with 0, 250, and 1,000μlL^−1^ ethanol, respectively. However, the POD activity of *B. cinerea*-inoculated blueberries treated with 1,000μlL^−1^ ethanol was greater than that of the other groups on day 0 (0.39Ug^−1^) but decreased rapidly and was significantly lower than that of *B. cinerea*-inoculated control group after 8days.

**Figure 6:**
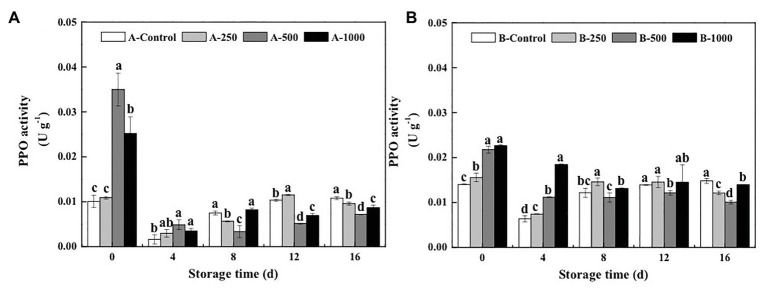
Effect of ethanol vapor on the polyphenol oxidase (PPO) activity of blueberries inoculated with **A** (*A. alternata*, A) and **B** (*B. cinerea*, B). Two-hundred and fifty, 500, and 1,000 are the amount of ethanol used (μlL^−1^). Error bars represent the standard deviation of the means (*n*=3). Different letters on the same column represent significant differences (*p*<0.05).

Polyphenol oxidase activity of blueberries inoculated with *A. alternata* and *B. cinerea* first decreased during storage and then increased (Figure 6). On day 0, PPO activity of the *A. alternata*- and *B. cinerea*-inoculated blueberries was significantly increased by the 500μlL^−1^ (3.47 and 1.55 times, respectively) and 1,000μlL^−1^ (2.50 and 1.61 times, respectively) ethanol treatments and then decreased rapidly. On the 4th day of storage, ethanol had no effect on PPO activity of the *A. alternata*-inoculated blueberries, whereas PPO activity of the *B. cinerea*-inoculated blueberries treated with 250, 500, and 1,000μlL^−1^ ethanol was 1.16, 1.74, and 2.88 times of the control, respectively. Furthermore, the PPO activity of the control blueberries increased rapidly after 4days of storage, whereas that of the blueberries treated with 500μlL^−1^ ethanol increased slowly but remained the lowest, with activities that were 33.5–55.2 and 8.7–31.9% lower than the control for the *A. alternata*- and *B. cinerea*-inoculated blueberries, respectively.

### Effect of Ethanol Vapor on Blueberry Cell Microstructure

**Figure 7:**
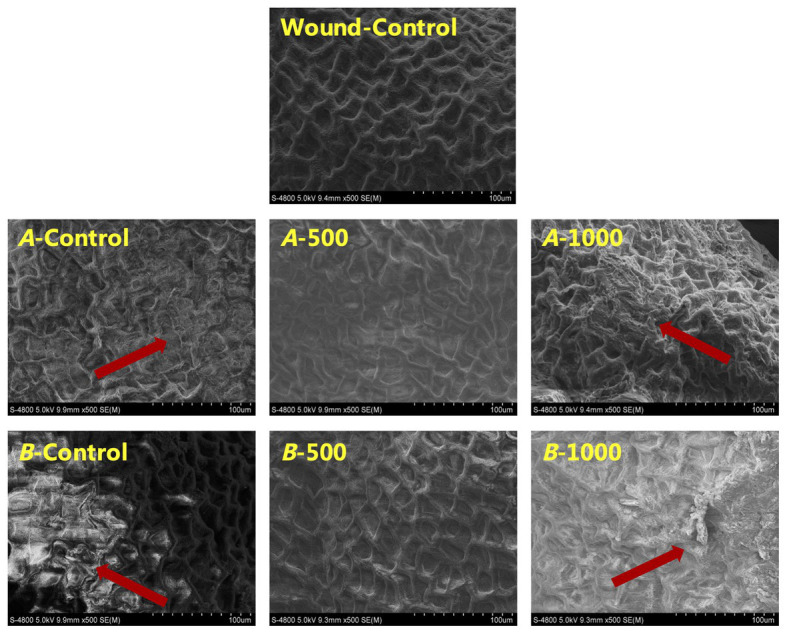
Scanning electron microscope (SEM) images of fruit cells of *A. alternata*- and *B. cinerea*-inoculated blueberry after treatment with ethanol on the 8th day of storage at 0±0.5°C. The scale bars represent 100μm.

Scanning electron microscope revealed that the pericarp cells of all inoculated blueberries were damaged to varying degrees (Figure 7). The 1,000μlL^−1^ ethanol-treated blueberry exhibited the greatest damage, whereas the pericarp cells of the 500μlL^−1^ ethanol-treated blueberry were the most similar to those of the Wound-Control, uniform, and intact.

**Figure 8:**
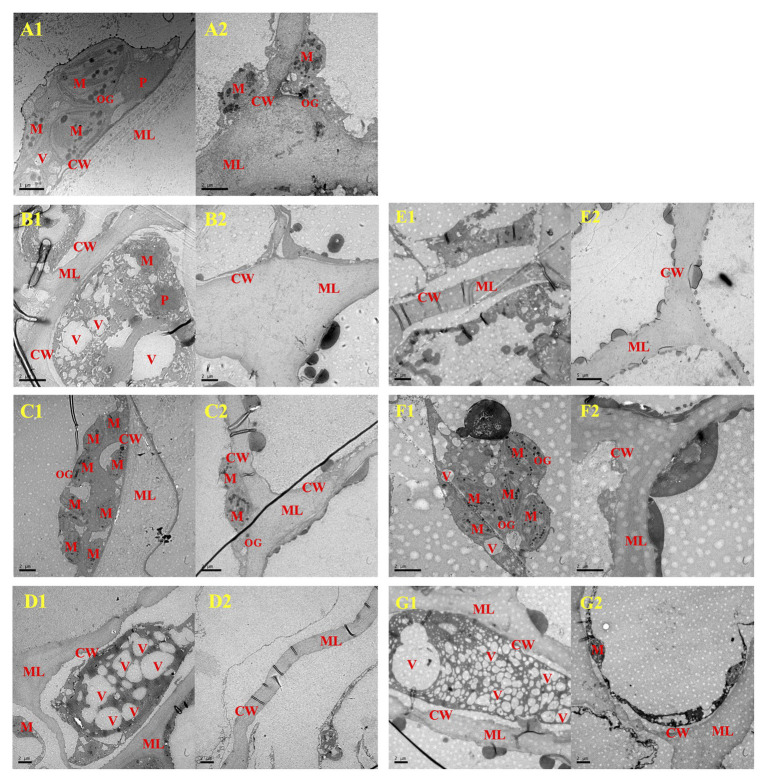
The cellular ultrastructure of *A. alternata*- **(B–D)** and *B. cinerea*- **(E–G)** inoculated blueberry fruit after treatment with ethanol on the 8th day of storage at 0±0.5°C. **A1** and **A2**, Wound-Control; **B1** and **B2**, *A*-Control; **C1** and **C2**, *A*-500; **D1** and **D2**, *A*-1000; **E1** and **E2**, *B*-Control; **F1** and **F2**, *B*-500; **G1** and **G2**, *B*-1000. CW, cell wall; M, mitochondria; ML, middle lamella; P, plastid; V, vacuole. The scale bars for **A1** represents 1μm, for **(A2,B1,B2,C1,C2,E1,F1,F2,G1,G2)** represent 2μm, and for **E2** represents 5μm.

Meanwhile, TEM revealed that pathogen inoculation induced conspicuous plasmolysis, and obscure membrane structure and broken intracellular compartmentalization (Figures 8B,E). However, the 500μlL^−1^ ethanol treatment appeared to protect cell structure and prevent plasmolysis (Figures 8C,F), with the complete nucleus, numerous complete mitochondria with typically closely arranged cristae, and osmiophilic granules dispersed in plastid matrix. However, numerous vacuoles were produced in cells of the 1,000μlL^−1^ ethanol-treated blueberries, and the vacuoles degraded the organelles (e.g., mitochondria and chloroplasts), thereby causing serious plasmolysis (Figures 8D,G).

## Discussion

Blueberries are vulnerable to microbial infection and rot throughout their production chain, from harvest to transportation, storage, and sale, thereby affecting their market value. However, the secondary plant metabolite ethanol exhibits strong and broad-spectrum antimicrobial activity and has been widely used for controlling fruit and vegetables diseases, owing to its safety, environmental friendliness, lack of residue, low cost, and widespread availability. Previous studies have reported that low levels of ethanol can be used to inhibit the growth of *Verticicladiella abietina*, *Penicillium citrinum*, *Trichoderma viride*, and *C. acutatum in vitro* (Wang et al., 2011, 2015), and Lichter et al. (2002) even reported that proved that ethanol liquid treatment could inhibit the spore germination and growth of *B. cinerea*. In the present study, the inhibition effect of ethanol vapor on the growth of fungal blueberry pathogens was assessed. Ethanol vapor treatment markedly reduced the growth of *A. alternata* and *B. cinerea* mycelia in a dose-dependent manner (Figure 1), which confirmed its effectiveness as a fungal growth inhibitor and may explain the previously reported effects of ethanol vapor on the decay of postharvest blueberries (Ji et al., 2019). Interestingly, *A. alternata* and *B. cinerea* differed in their sensitivity to ethanol. For example, *B. cinerea* was inhibited in a dose-dependent manner (1,000μlL^−1^ >500μlL^−1^ >250μlL^−1^), whereas *A. alternata* was most inhibited by the 500μlL^−1^ ethanol treatment, in contrast to previous reports – the higher the ethanol concentration, the stronger the ability of killing *B. cinerea* spores (Lichter et al., 2002; Karabulut et al., 2004a,b). The difference for *A. alternata* with previous studies could be due to differences in ethanol treatment methods (e.g., liquid treatment vs. vapor treatment), which could have different modes of action or cause different damage to microbial cells. Ethanol vapor is reportedly useful for controlling the postharvest decay of fruit and vegetables, such as sweet cherries (Karabulut et al., 2004a), table grapes (Lichter et al., 2002; Karabulut et al., 2004b), loquats (Wang et al., 2015), and blueberries (Ji et al., 2019), which can be explained by the inhibitory activity of ethanol against the growth of rot fungi of many fruit and vegetables.

However, it was found that the disease control effect of ethanol on postharvest fruit and vegetables relies both on its direct antimicrobial properties, as well as on the mechanism of “priming,” which induces and improves the resistance of fruit and vegetables to postharvest diseases (Tzortzakis, 2010). The activation of defense-related enzymes, such as GLU, CHI, PAL, POD, and PPO, in fruit and vegetables is considered to be an important factor in the diseases resistance of postharvest horticultural crops (Tian et al., 2006; Wang et al., 2014; Shao et al., 2015). In the present study, the activity of both CHI and GLU, which can degrade chitin and *β*-1,3-glucan in fungal cell walls and effectively prevent the infection of fruits and vegetables, was significantly greater in ethanol-treated blueberries than in control blueberries (Souza et al., 2017; Figures 2, 3). Meanwhile, PAL and POD are important resistant substance synthetases in fruit and vegetables and can catalyze the synthesis of lignin, flavonoids, and phenols. This is important because phenols can be transformed into quinones, which are more toxic to pathogens, and because lignin is valuable for strengthening fruit cell walls and, thereby, inhibiting pathogenesis (Farkas and Stahmann, 1996). In the present study, both PAL and POD activities were significantly enhanced by the 500μlL^−1^ ethanol treatment (Figures 4, 5). PPO is a copper enzyme that also contributes to the lignification of host cells and catalyzes the oxidation of phenolic compounds into toxic quinones with strong antifungal activity against pathogens (Cindi et al., 2016). In the present study, the PPO activity of blueberry increased immediately after the ethanol vapor treatment, potentially enhancing resistance of blueberries against pathogens. Therefore, these effects could collectively contribute to the development of disease resistance in blueberries against *A. alternata* and *B. cinerea*. Similarly, many studies also have been reported on enhancing disease resistance of fruit and vegetables by inducing the activities of DREs, such as in pear fruit (GLU, CHI, PAL, POD, and PPO) with *L*-glutamate treatment against *Penicillium expansum* (Jin et al., 2019), in pear fruit (GLU, CHI, PAL, POD, and PPO) with *γ*-aminobutyric acid treatment against *P. expansum* (Yu et al., 2014), in tomato fruit (PAL, PPO, CHI, and GLU) with *L*-arginine treatment against *B. cinerea* (Zheng et al., 2011), and in strawberry (CHI and GLU) with tea tree oil and hot air treatment against *B. cinerea* (Wei et al., 2018). Therefore, the induction of disease resistance by enhancing DREs (CHI, GLU, PAL, POD, and PPO) activities might be an important mechanism by which ethanol vapor is able to reduce the postharvest decay of blueberries. Ethanol vapor can induce the disease resistance of blueberries infected by *A. alternata* and *B. cinerea*, but the disease resistance of blueberry infected by *B. cinerea* is stronger than that of blueberry infected by *A. alternata*.

**Figure 9:**
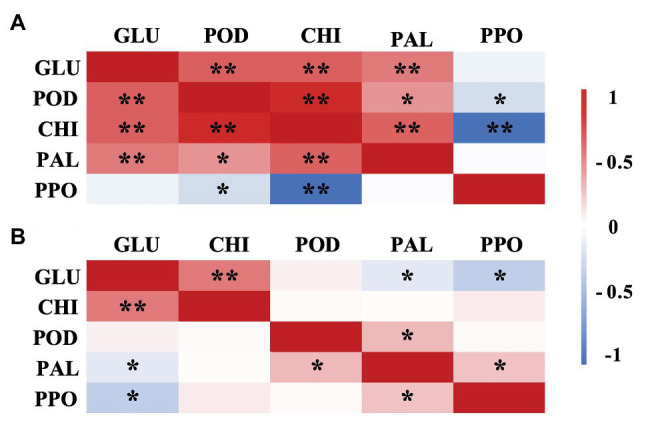
Pearson correlation matrix of the disease resistance-related enzyme activities (GLU, CHI, PAL, POD, and PPO) in ethanol-treated blueberries inoculated with *A. alternata* **(A)** and *B. cinerea* **(B)**. ^*^Significance at the *p*<0.05 probability level. ^**^Significance at the *p*<0.01 probability level.

Correlation analysis indicated that there were different correlations among the DREs (GLU, CHI, PAL, POD, and PPO) in *A. alternata*- and *B. cinerea*-inoculated blueberries with ethanol treatment (Figure 9), which indicated that different pathways were involved in the disease resistance-promoting effects of ethanol against different pathogens. For example, PPO activity was negatively or not correlated with GLU, CHI, and POD activity in blueberries affected with either pathogen, possibly because the production of quinones or the other defense-related substances catalyzed by PPO is not one of the main mechanism underlying such ethanol-induced disease resistance. The PAL, the key enzyme of phenylpropanoid pathway, its activity in *A. alternata*-inoculated blueberries with ethanol treatment was significantly positively correlated with CHI and GLU, which indicated that ethanol vapor inducers could stimulate defense mechanisms, such as phenylpropanoid pathway activation, helping to protect the blueberries against *A. alternate*. Similar result was confirmed on strawberries treated by terpinen-4-ol (Li et al., 2020).

In addition, the cells microstructure of the 500μlL^−1^ ethanol-treated blueberries was the most similar to that of the control (Wound-control) in the present study, being uniform and intact (surface structure; Figure 7), and exhibited the relatively abundant inclusions (ultrastructure; Figure 8). These findings indicated that the senescence of blueberry cells could be delayed by appropriate ethanol vapor treatment. The 500μlL^−1^ ethanol treatment also resulted in the attachment of rough substances to the closely arranged fiber microfibrils on the cell walls, and those substances could be phenolic substances and lignin that was induced by the ethanol treatment, thereby improving the firmness of the cell wall and the barrier defense capability, thus, the pathogen resistance. However, further study on ethanol vapor inducing disease resistance of blueberries is needed.

## Data Availability Statement

The original contributions presented in the study are included in the article/supplementary material, further inquiries can be directed to the corresponding author.

## Author Contributions

YJ, WH, ZX, and AJ: conceptualization. WH: methodology, project administration, and funding acquisition. YJ: validation, formal analysis, data curation, and writing-original draft preparation. YJ and JL: investigation. YJ, WH, JL, ZX, AJ, XY, YG, KF, and GS: writing-review and editing. WH and ZX: supervision. All authors contributed to the article and approved the submitted version.

### Conflict of Interest

The authors declare that the research was conducted in the absence of any commercial or financial relationships that could be construed as a potential conflict of interest.
